# QTL mapping of antixenosis resistance to common cutworm (*Spodoptera litura* Fabricius) in wild soybean (*Glycine soja*)

**DOI:** 10.1371/journal.pone.0189440

**Published:** 2017-12-12

**Authors:** Nobuhiko Oki, Akito Kaga, Takehiko Shimizu, Masakazu Takahashi, Yuhi Kono, Motoki Takahashi

**Affiliations:** 1 Kyushu Okinawa Agricultural Research Center,National Agriculture and Food Research Organization, Suya, Koushi, Kumamoto, Japan; 2 Institute of Crop Science, National Agriculture and Food Research Organization, Kannondai, Tsukuba, Ibaraki, Japan; Aberystwyth University, UNITED KINGDOM

## Abstract

The common cutworm (CCW; *Spodoptera litura* Fabricius) is a serious herbivorous insect pest of soybean (*Glycine max*) in Asia and Oceania. Previously, we identified quantitative trait loci (QTLs) for CCW-antibiosis-resistance, *CCW-1* and *CCW-2*, and antixenosis-resistance, *qRslx1* and *qRslx2*, in the cultivar ‘Himeshirazu’. The effects of these QTLs are useful in the breeding of CCW-resistant cultivars. In this study, we conducted an antixenosis bioassay on CCW using recombinant inbred lines derived from a cross between a wild soybean (*Glycine soja*) and the leading cultivar ‘Fukuyutaka’ to identify CCW-resistance genes in *G*. *soja*. The QTL analysis revealed six and four novel antixenosis-resistance QTLs in 2012 and 2013, respectively. Among them, the QTLs on chromosomes 2 and 7, designated *qRslx4* and *qRslx3*, respectively, were stably detected in both years. *qRslx3* exhibited the largest effect in both years, suggesting that *qRslx3* can be exploited in the breeding of CCW-resistant soybean. Furthermore, *qRslx3* and *qRslx4* can be used, along with previously reported QTLs from ‘Himeshirazu’, to enhance the CCW-resistance of soybean cultivars because their chromosomal positions are unique. These new CCW-resistance QTLs from *G*. *soja* should play important roles in the breeding of CCW-resistant soybean cultivars.

## Introduction

The common cutworm (CCW, *Spodoptera litura* Fabricius), which is an insect pest of many important crops in Asia and Oceania, feeds on the leaves of more than 100 plant species [1] and causes serious yield losses to soybean (*Glycine max*). The development of cultivars with CCW resistance would reduce insecticide applications and stabilize soybean production. However, the breeding of CCW-resistant soybean cultivars has been unsuccessful for many years. The phenotyping of resistance under field conditions is unreliable because the extent of the feeding damage is largely affected by the distance from plants on which the CCW had ovipositioned and because other insect species cause feeding damage. In addition, the resistant germplasm used for the breeding have undesirable agronomic traits, which have made it difficult to breed resistant cultivars with high yield and quality levels. Marker-assisted selection (MAS) is expected to be a useful tool for CCW-resistance breeding in soybean because breeders can select lines containing resistance genes without performing phenotypic evaluations. To exploit MAS in the breeding of CCW-resistant cultivars, a genetic analysis is necessary to develop markers associated with resistance genes.

The resistance mechanisms to herbivorous insects are generally divided into antixenosis (affecting feeding behaviors of insect pests; non-preference mechanisms) and antibiosis (having detrimental effects on development), and genetic analyses have been conducted to identify resistance genes to herbivorous insects. Three Japanese soybean landraces, ‘PI171451’, ‘PI227687’ and ‘PI229358’ were initially identified resistant to the Mexican bean beetle (*Epilachna varivestis* Mulsant) [2]. Subsequently, quantitative trait loci (QTLs) for antixenosis and antibiosis resistance to corn earworm (*Helicoverpa zea* Boddie), an herbivorous insect of soybean in the USA, were reported in the same three landraces [3–5]. In particular, both antixenosis- and antibiosis-resistance-related QTLs to corn earworm were located on chromosome (Chr) 7, in earlier linkage group (LG) M. Interestingly, the QTLs are effective against soybean looper (*Pseudoplusia includens*) and tobacco budworm (*Heliothis virescens*) [6–8]. Additionally, the QTL detected on Chr 15 (LG-E) has major effects in two different recombinant-inbred line (RIL) populations [9]. A QTL analysis for CCW-resistance revealed three major QTLs for antibiosis, *qCCW6_1*, *qCCW10_1* and *qCCW12_2*, and three major QTLs for antixenosis, *qCCW10_1*, *qCCW10_2* and *qCCW12_1*, in the RIL populations derived from Chinese soybean germplasms [10]. Furthermore, 26 and 43 antibiosis-resistance QTLs for CCW were detected in 2009 and 2011, respectively, using association analyses [11]. Although these reports revealed many antibiosis- and antixenosis-resistance QTLs, the resistance mechanisms associated with these genes are still unresolved.

A forage soybean cultivar, ‘Himeshirazu’ (PI594177), exhibits clear resistance to CCW [12]. Two antibiosis-resistance QTLs, *CCW-1* and *CCW-2*, were detected on Chr 7 by a QTL analysis of an F_2_ population derived from a cross between the susceptible cultivar ‘Fukuyutaka’ (PI506675) and ‘Himeshirazu’ [13]. The resistance alleles of both loci originated from ‘Himeshirazu’. *CCW-1* and the resistance QTL to corn earworm on Chr 7 detected in PI229358 were proven to be identical by crossing tests [14]. However, an antixenosis-resistance analysis using RILs developed from the F_2_ population revealed two antixenosis-resistance QTLs [15]. QTLs on Chr 7 and Chr 12 (LG-H) were named *QTL for resistance to S*. *litura antixenosis1* (*qRslx1*) and *qRslx2*, respectively. The resistance allele of *qRslx1* was derived from ‘Himeshirazu’, whereas that of *qRslx2* was from ‘Fukuyutaka’. The location of *qRslx1* is almost the same as that of *CCW-1*, suggesting that the same gene provides both antibiosis and antixenosis resistance. We introduced *CCW-1* (*qRslx1*), *CCW-2* or both into ‘Fukuyutaka’ to develop near-isogenic lines (NILs) and confirmed the effects of these genes [14]. Under field conditions, *CCW-1* NILs suppress CCW larval accumulation [16].

Although *CCW-1* (*qRslx1*) and *CCW-2* are effective, the resistance level of the NIL containing both resistance genes is still lower than that of ‘Himeshirazu’ [14, 16]. Thus, we focused on CCW resistance in wild soybean (*Glycine soja*) as a new resistance gene source. *G*. *soja* is an annual plant that is found in eastern and northeastern China, Japan, Korea and far eastern Russia [17]. In Japan, *G*. *soja* is distributed broadly in disturbed habitats, such as riverbanks, roadsides and at the edges of fields [18–21]. To our knowledge, no reports on the CCW resistance of *G*. *soja* exist. We empirically know that *G*. *soja* is highly resistant to herbivorous insects and that defoliated areas of *G*. *soja* are smaller than those of soybean when collected germplasm is grown. The objectives of this study were (1) to assess the CCW-antixenosis resistance of *G*. *soja* and (2) to identify novel CCW-antixenosis-resistance genes using RILs derived from a cross between *G*. *soja* and ‘Fukuyutaka’.

## Materials and methods

### Evaluation of antixenosis resistance to CCW

**Fig 1:**
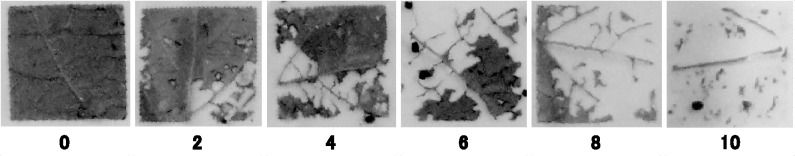
Examples of the leaflet defoliation ratings of soybean. The numbers below the pictures are the defoliation values (on a scale of 0 to 10) for each leaflet segment.

A set of 202 RILs were developed from an F_2_ population derived from a cross between *G*. *soja* (NIAS Genebank accession JP110755) collected in Hiroshima Prefecture in southern Japan and ‘Fukuyutaka’ (NIAS Genebank accession JP29668), which is a leading cultivar in southwestern Japan and is susceptible to CCW. The F_7_ and F_8_ RILs and their parents were grown in a field (Andosol soil) at the Kyushu Okinawa Agricultural Research Center (located at 32°52′N, 130°44′E) in 2012 and 2013. The planting dates were 26 June 2012 and 25 June 2013. The inter-row spacing and hill spacing were 70 cm and 42 cm, respectively. Three individuals were grown for each RIL, and samplings of leaflets were conducted equally. The flowering dates of the RILs were recorded when flowering of two individuals were observed. Stakes were used to support each plant because *G*. *soja* and the RILs have long stems. Approximately three stems per plant were guided to the stakes, and other stems were cut because they may twine to the stakes of other lines. No pesticides were applied over the experimental period. The procedure for the bioassay of antixenosis was almost the same as previously reported [15]. Briefly, an antixenosis test was performed in an air-conditioned room maintained at 23.5 ± 1°C with a 12-h light/12-h dark photoperiod. The bioassay was performed in Petri dishes, with the bottom of each dish being covered with a moist filter paper. Fully expanded leaflets of a similar age were collected and cut into square segments of approximately 25 mm × 25 mm. A standard leaflet segment of ‘Akisengoku’ and a test leaflet segment of one of the RILs, or a parent, were placed with their abaxial sides facing upward on the filter paper. ‘Akisengoku’ was used as a standard cultivar for comparison in the feeding tests conducted with the RIL, because it exhibits intermediate antixenosis-resistance between those of ‘Fukuyutaka’ and *G*. *soja*. Third-instar CCW larvae that had been reared on an artificial diet (Insecta LF S; Nippon Nousan Kougyo Co., Yokohama, Japan) were used for the bioassay. A third-instar CCW larva was placed into each petri dish on the filter paper between the leaflet segments. Approximately 14 h later, visual defoliation was assessed and rated on a scale of 0 to 10 for the two leaflet segments (Fig 1). A rating of 0 implied that the leaflet segment was not defoliated, while a rating of 10 implied that the leaflet segment was fully defoliated. The antixenosis-resistance levels of each RIL and the parents were evaluated using 12 and 72 leaflet segments, respectively. The following formula was used to calculate the antixenosis index (*C*), which was used to compare the test plants with the standard plant [22]:

C=2∑A/(∑M+∑A), (1)

where *A* = the defoliation rate of the sample leaf segment and *M* = the defoliation rate of the standard leaf segment (‘Akisengoku’). A *C* value was calculated using 12 leaflet segments. A *C* value of 1 implied that the feeding on the test plant was the same as the feeding on the standard plant. A *C* value > 1 implied a preference for the test plant, and a *C* value < 1 implied that the test plant had a greater antixenosis-resistance than the standard cultivar.

### Genotyping of markers

Total genomic DNA was extracted from young fresh leaves (0.3 g) at the vegetative growth stage according to the procedure of Khosla et al. [23] with minor modifications. A total of 163 genome-wide single nucleotide polymorphism markers (S1 Table) were selected based on the genotype information and analyzed as described by Kaga et al. [24] using the MassARRAY system (Agena Bioscience, San Diego, CA, USA). In addition, 236 simple sequence repeat (SSR) markers (S1 Table) were selected based on the F_2_ linkage map of the same cross combination and analyzed as described by Kuroda et al. [25].

### Linkage and QTL analyses

A genetic linkage map was constructed using JoinMap 4.0 [26]. The logarithm-of-odds threshold for a linkage grouping was 4.0, and the marker order was determined using a maximum likelihood mapping algorithm. The recombination frequencies were converted into genetic distances (cM) using the Haldane mapping function. A QTL analysis was conducted with the mean values of the RILs using the software package MultiQTL ver. 2.6 as described by Kaga et al. [27]. Briefly, a single QTL model was fit for each trait–chromosome (linkage group) combination. Chromosomal statistical significance thresholds (α = 0.05) for putative QTLs were tested by 10,000 runs of a permutation test [28]. Multiple interval mapping [29] was then conducted to reduce the background variation, taking into account QTL effects from other chromosomes. After the permutation test runs, the parameters of significant QTLs (statistical thresholds α = 0.05) were reported as position, substitution effect, and percentage of variance explained (PVE). RILs were classified into two groups based on the genotypes of the detected QTLs, and a one-way analysis of variance was conducted using PASW Statistics ver. 18 to compare the mean *C* values.

## Results

**Fig 2:**
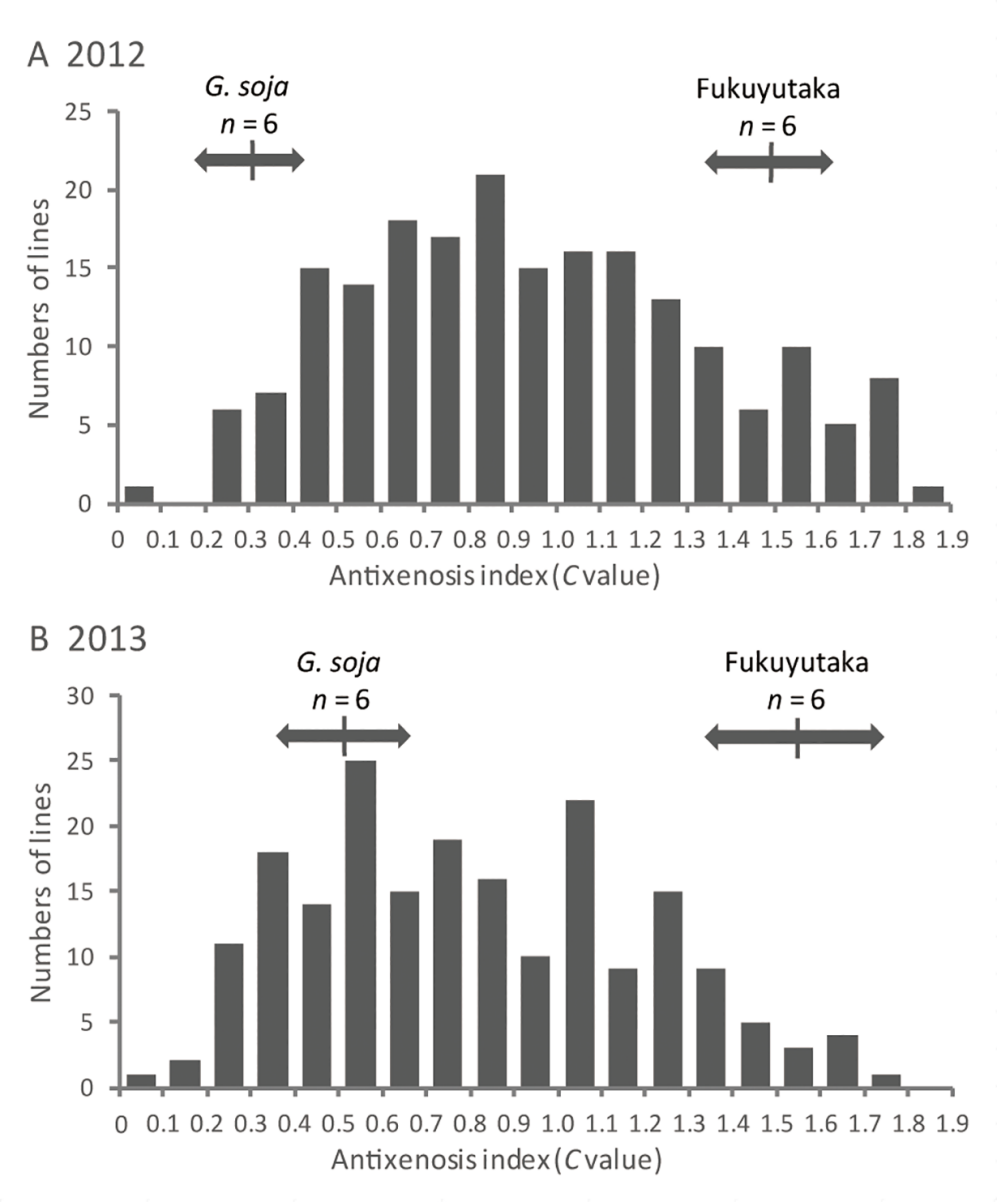
**Frequency distributions of the antixenosis indices of the recombinant-inbred lines derived from a cross between *Glycine soja* and ‘Fukuyutaka’ in 2012 (A) and 2013 (B).** The antixenosis resistance was evaluated using *C* values (calculated using Equation 1), which represent the resistance relative to that of a standard cultivar, ‘Akisengoku’. Arrows and vertical lines represent the positions of the standard deviations and mean values of the parents, respectively.

Antixenosis-resistance was evaluated using a preference comparison between the test line and a standard cultivar, ‘Akisengoku’. The average *C* values of *G*. *soja* and ‘Fukuyutaka’ were 0.29 and 1.57, respectively, in 2012, and 0.50 and 1.54, respectively, in 2013 (Fig 2). The *C* values of *G*. *soja* were significantly lower than those of ‘Fukuyutaka’ in both years (*p* < 0.001). The frequency distributions of the 202 RILs’ *C* values were continuous and extended beyond the ranges of the parents in both years (Fig 2). The ranges of the RILs’ *C*-values were 0.05 to 1.81 in 2012, and 0.07 to 1.80 in 2013, respectively. The mean *C*-values of the RILs were 0.95 and 0.82 in 2012 and 2013, respectively. *C*-values of 3.0% and 23.1% for the RILs were smaller than those of *G*. *soja*, and *C*-values of 9.0% and 3.0% were larger than those of ‘Fukuyutaka’ in 2012 and 2013, respectively. The broad-sense heritability of antixenosis-resistance was estimated as 90.2 and 80.2%, in 2012 and 2013, respectively. A significant correlation was detected between the *C* values of 2012 and 2013 (*r* = 0.57, *p* < 0.001).

We constructed a linkage map of the RILs using the segregation data for 399 loci in 202 RILs. The total map length was 2,376 cM (S1 Fig) and the average and maximum marker distance were 6.3 cM and 35.1 cM, respectively. In total, 37 marker loci (9.3%) showed segregation ratios significantly (*p* ≤ 0.05) deviated from the expected 1:1 ratio of *G*. *soja* and Fukuyutaka homozygote. Most markers with segregation distortion were identified at the distal ends of chromosomes. Severe segregation distortion (*p* ≤ 0.01) was observed for the markers on Chr 4 (14 cM), Chr 6 (37 cM and 169 cM), Chr 8 (7 cM) and Chr18 (71 cM) (S1 Table). The genotype frequency of ‘Fukuyutaka’ near these regions was increased.

Six and four QTLs associated with *C* values were detected in 2012 and 2013, respectively (Table 1). The resistance alleles of these QTLs were derived from *G*. *soja*, except the two QTLs detected on Chr 9 (LG-K) and Chr 16 (LG-J) in 2012. The substitution effects of *C* values and the PVEs of the resistance QTLs derived from *G*. *soja* ranged from −0.384 to 0.182 and from 3.1 to 24.7, respectively. Because the QTLs on Chr 2 (LG-D1b) and Chr 7 were detected in both years, we have provisionally designated these QTLs as *QTL for resistance to S*. *litura antixenosis 4* (*qRslx4*) and *qRslx3*, respectively. *qRslx3* had the greatest substitution effect and PVE in both years. We also conducted a QTL analysis for days to flowering (S2 Table, S2 Fig). QTLs for flowering time were detected on Chr 16 and 19 (LG-L) near the QTLs for antixenosis-resistance in 2012.

**Table 1:** Quantitative trait loci (QTLs) associated with the antixenosis index (*C* value) detected in the recombinant-inbred lines derived from *Glycine soja* and ‘Fukuyutaka’.

Year	Chr	QTL name	LOD^a^	PEV^b^	Substitution effect^c^	Peak position (cM^d^)	QTL region (cM)
2012	2	*qRslx4*	2.7	4.9	-0.173	136.9	Satt459 (127.3)—GMES2618 (144.4)
2012	7	*qRslx3*	7.6	12.8	-0.281	3.4	GMSC514 (0.0)—Satt150 (9.0)
2012	9		4.1	5.4	0.182	35.1	C09-BARC-015513-01990 (34.2) -Satt055 (35.4)
2012	14		5.8	8.5	-0.229	38.2	C14-BARC-044549-08718 (30.1)—Satt416 (39.1)
2012	16		2.8	4.3	0.163	20.0	C16-BARC-016775-02320 (16.0)—s016200713 (23.5)
2012	19		5.2	9.9	-0.247	75.0	Satt561 (68.4)—Sat_286 (82.8)
2013	2	*qRslx4*	2.8	4.5	-0.163	125.4	C02-BARC-026065-05239 (120.5)—Satt274 (127.2)
2013	6		5.6	7.9	-0.217	124.1	sF3H (122.6)—Satt708 (126.8)
2013	7	*qRslx3*	11.8	24.7	-0.384	4.1	GMSC514 (0.0)—Satt150 (9.0)
2013	18		2.2	3.1	-0.136	49.4	Satt352 (43.4)—Satt505 (50.9)

^a^ Logarithm-of-odds.

^b^ Percentage of variance explained.

^c^ Substitution effect of the *G*. *soja* allele. A negative value implies that the *G*. *soja* allele had a resistance effect.

^d^ Centimorgan.

**Fig 3:**
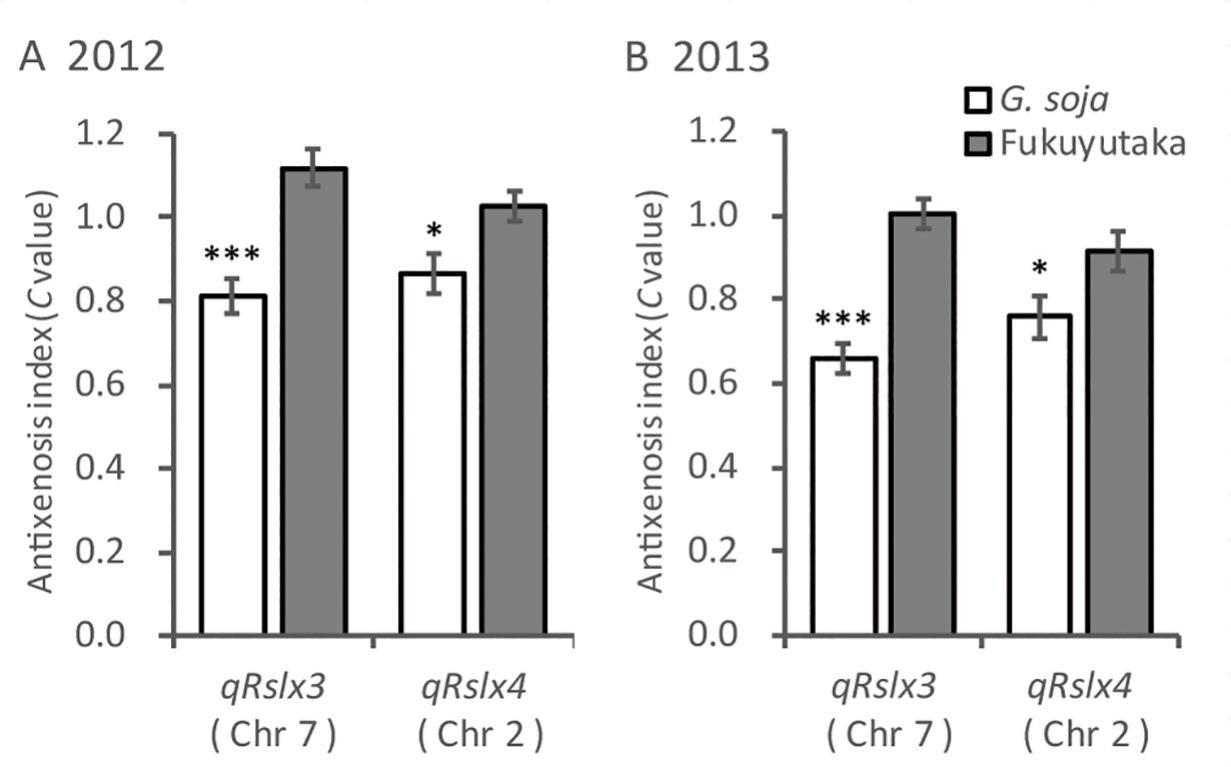
**Differences in the mean antixenosis (*C*) values of the *Glycine soja* × ‘Fukuyutaka’-derived recombinant-inbred lines for each genotype in (A) 2012 and (B) 2013.** Each genotype of *qRslx3* and *qRslx4* is represented by markers in the quantitative trait loci regions (Table 1). Values represent means ± standard errors. *, *** Significant at the 0.05 and 0.001 levels, respectively.

**Fig 4:**
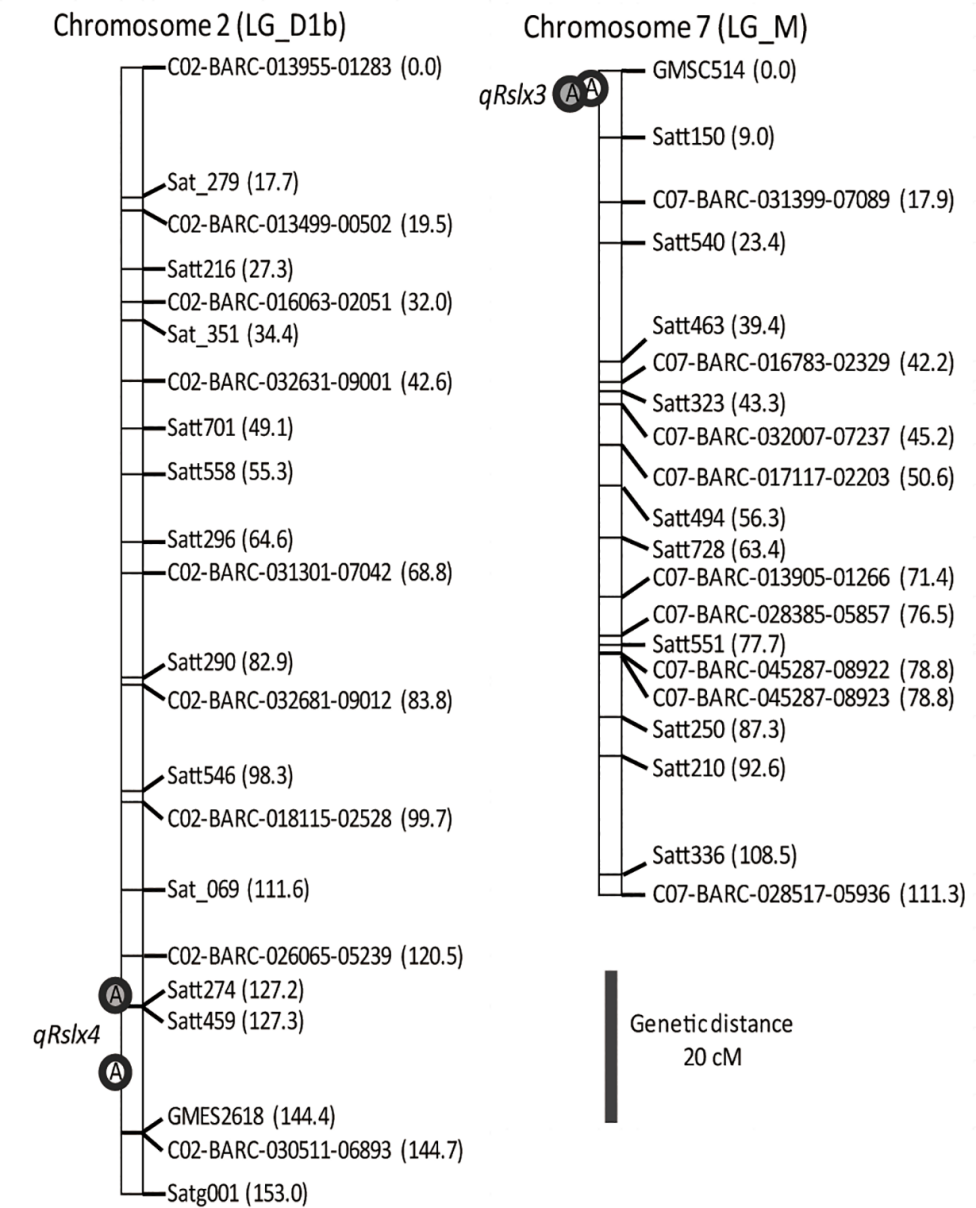
Location of the quantitative trait loci (QTLs) for antixenosis-resistance, *qRslx4* on chromosome 2 (left) and *qRslx3* on chromosome 7 (right) in *Glycine soja* × ‘Fukuyutaka’-derived recombinant-inbred lines. Labels to the right of the bars show the marker names. The white and gray circles represent the locations of the antixenosis-resistance QTLs observed in 2012 and 2013, respectively.

To clarify the effects of *qRslx3* and *qRslx4*, RILs were classified into two genotype-based groups, homozygous for *G*. *soja* and homozygous for ‘Fukuyutaka’, using markers located on the both sides of each QTL (Fig 3). To simplify the analysis, data from RILs that were heterozygous at the QTL regions or had undergone recombination in the QTL regions were excluded. The markers used to classify the RILs are listed in Table 1. *qRslx4* was detected on different, but close, intervals on Chr 2 in 2012 and 2013 (Fig 4, Table 1). The markers, C02-BARC-026065-05239 and GMES2618, covering both intervals, were used to classify the genotypes of RILs of *qRslx4*. A one-way analysis of variance revealed that *qRslx3* and *qRslx4* exhibited significant effects on the *C* values of the RILs (Fig 3).

## Discussion

We compared the antixenosis-resistance of *G*. *soja* and ‘Fukuyutaka’ by evaluating the *C* values, revealing that *G*. *soja* possessed a greater antixenosis-resistance than ‘Fukuyutaka’ (*p* < 0.001, Fig 2). Thus, *G*. *soja* possesses resistance gene(s) or allele(s) that can enhance the CCW resistance of soybean. The frequency distributions for the *C* values of the RILs derived from the cross between *G*. *soja* and ‘Fukuyutaka’ were continuous in both years, suggesting that the *C* value is quantitatively controlled by multiple loci. To elucidate the genetic basis for the CCW-antixenosis-resistance of *G*. *soja*, we constructed a genetic linkage map for the RILs and conducted a QTL analysis. The total length of the linkage map was 2,376 cM (S1 Fig) which is comparable with the 2,524 cM and 2,383 cM lengths of the linkage maps developed by Song et al. [30] and Liu et al. [31], respectively. The order of the markers on the linkage map generally agreed with the positions on the genome sequence of Glyma1 [32], with exceptions for inversions among several markers on the top and bottom of Chr 5 (LG-A1) and the top of Chr 13 (LG-F) (S1 Table). Six and four QTLs for antixenosis-resistance were detected in 2012 and 2013, respectively. Among them, a *G*. *soja* allele on *qRslx3* had the greatest substitution effect on the *C* values and the PVEs in both years (Fig 4, Table 1). *qRslx3* could be useful for breeding because it is stable and the most effective *G*. *soja* resistance allele against CCW. As described above, the CCW-resistance genes *CCW-1* (*qRslx1*) and *CCW-2* were identified on Chr 7 [13, 15]. A resistance gene to corn earworm was reported at almost the same position as *CCW-1* (*qRslx1*) [3–5]. In contrast, the position of *qRslx3* is clearly different from those of *CCW-1* (*qRslx1*) and *CCW-2* because *qRslx3* is separated by approximately 35 cM and 15 cM, respectively. Thus, *qRslx3* can be exploited as a new source of CCW resistance to enhance soybean lines that also possess *CCW-1* (*qRslx1*) and *CCW-2*. Additionally, an antibiosis-resistance QTL to CCW, *qCCW7_1*, was detected from a Chinese soybean germplasm on Chr 7, and reported to be close to *CCW-2* [10]. It is difficult to determine whether *qRslx3* and *qCCW7_1* are identical, because of the lack of common markers in their linkage maps. Recently, an association analysis revealed an antibiosis-resistance QTL to CCW near the SSR marker GMES3041 on Chr 7 [11]. Based on the physical positions of the primer sequences, the marker (14.9 Mb) is located on the interval between C07-BARC-032007-07237 (13.6 Mb) and C07-BARC-017117-02203 (15.4 Mb) in our linkage map; therefore, the QTL is clearly different from *qRslx3*. Thus, Chr 7 is an important chromosome for insect resistance to soybean, harboring many resistance genes. Because *qRslx3* is expected to play an important role in the breeding of CCW-resistant cultivars, more detailed analyses are necessary to reveal the precise position and degree of the antixenosis effect.

Another significant QTL, *qRslx4*, was detected on the bottom of Chr 2 in both years (Fig 4, Table 1). The PVE and the substitution effect of the *G*. *soja* allele on *qRslx4* were less than those of *qRslx3* (Fig 3). Although a two-way ANOVA was conducted to elucidate the epistatic effect between *qRslx3* and *qRslx4*, no significant epistatic effect was detected in either year. An antixenosis-resistance QTL to corn earworm was detected on Chr 2 in the region between SSR markers Satt290 and Satt141 [3, 33]. The estimated linkage distance between the antixenosis-resistance QTL to corn earworm on Chr 2 and *qRslx4* was determined to be approximately 30 cM with the aid of Soybase (http://www.soybase.org/), indicating that these QTLs are not derived from the same gene. Similarly, an antibiosis-resistance QTL to CCW, *qCCW2_1*, was detected on Chr 2 from a Chinese soybean germplasm [10]. However, *qRslx4* and *qCCW2_1* are probably not the same gene because *qCCW2_1* exhibits only epistatic effects and no QTLs with additive effects was detected on Chr 2. Furthermore, an association analysis of antibiosis-resistance to CCW in a Chinese soybean germplasm population revealed four antibiosis-resistance QTLs to CCW on Chr 2 [11]. The QTL detected at Sat_289 and *qRslx4* may be the same gene because the estimated distance between the flanking markers of these QTLs was approximately 10 cM. Two QTLs for resistance to bean pyralid (*Lamprosema indicata* Fabricius), *BP2-1* and *BP2-2*, were reported on Chr 2 [34]. These QTLs are clearly different from *qRslx4* because the estimated linkage distances were more than 60 cM. We evaluated the effects of *qRslx4* and revealed relationships with previously reported resistance QTLs. These could be used to enhance the CCW resistance of soybean cultivars.

The QTLs on Chr 9, 14, 16 and 19 were detected only in 2012, and the QTLs on Chr 6 and 18 were detected only in 2013. Resistance QTLs to CCW, corn earworm and bean pyralid have been reported on these six chromosomes and some may be common to other resources [4, 5, 10, 11, 34, 35]. Because the effects of these QTLs on antixenosis are unstable and relatively small (Table 1), it may be difficult to use them immediately in breeding programs. In addition, the effects of the QTLs on Chr 16 and 19 on antixenosis may be influenced by flowering time because the flowering time-associated QTLs were detected near these resistance QTLs (S2 Table). Thus, the effects of these QTLs must be confirmed by additional analyses. We are developing NILs containing each of these QTLs to elucidate their single effects.

Global warming will likely change the geographical distribution of insects [36], and high temperatures have been predicted to cause frequent outbreaks of lepidopteran insects [37]. The CCW will expand its habitat during global warming, causing more economic damage to soybean production. To our knowledge, this is the first report to detect CCW-resistance QTLs from *G*. *soja*. The resistance alleles originating from the genetically remote wild ancestor are likely to be novel, even though resistance QTLs were previously reported in close genomic regions. Thus, the resistance QTLs from *G*. *soja* and the associated markers are expected to allow for the efficient breeding of soybean cultivars with high levels of CCW resistance. We expect that soybean cultivars with greater CCW resistance can be developed by pyramiding *qRslx3* and *qRslx4* in addition to *CCW-1* (*qRslx1*) and *CCW-2*, and this will stabilize the soybean production in the face of continued global warming.

## Supporting information

S1 Fig A genetic linkage map constructed for recombinant-inbred lines between *G*. *soja* and Fukuyutaka. (PDF)

S2 Fig Frequency distributions of days to flowering for the recombinant-inbred lines derived from a cross between *Glycine soja* and ‘Fukuyutaka’ in 2012 (A) and 2013 (B). Days to flowering for ‘Fukuyutaka’ were 47 and 46 in 2012 and 2013, respectively. Days to flowering for *G*. *soja* were 67 and 69 in 2012 and 2013, respectively.
(PDF)

S1 Table Positions, segregation distortions and primer sequences of the single nucleotide polymorphism markers and simple sequence repeat markers used to construct the genetic linkage map of the RILs between *G*. *soja* and ‘Fukuyutaka’. (XLSX)

S2 Table All QTL information for *C* values and days to flowering detected in the RILs derived from *G*. *soja* and Fukuyutaka. (XLSX)
