# Supplementary figures and images for: QTL mapping of antixenosis resistance to common cutworm (Spodoptera litura Fabricius) in wild soybean (Glycine soja)
Source: PLoS One. 2017 Dec 12;12(12):e0189440. doi: 10.1371/journal.pone.0189440 (PMC5726720; doi:10.1371/journal.pone.0189440)

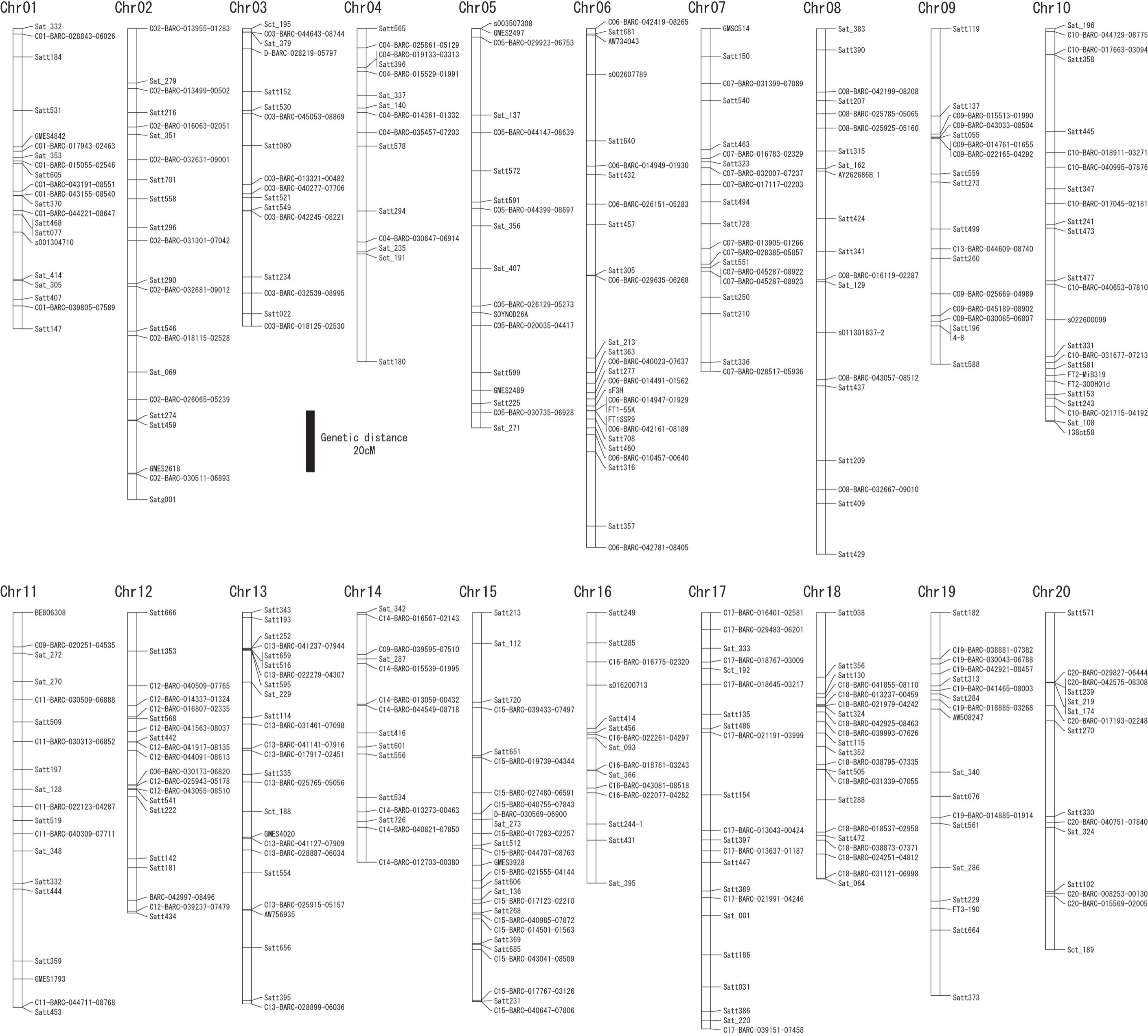

Supplement: S1 Fig — (PDF) [file pone.0189440.s001.pdf]

A 2012

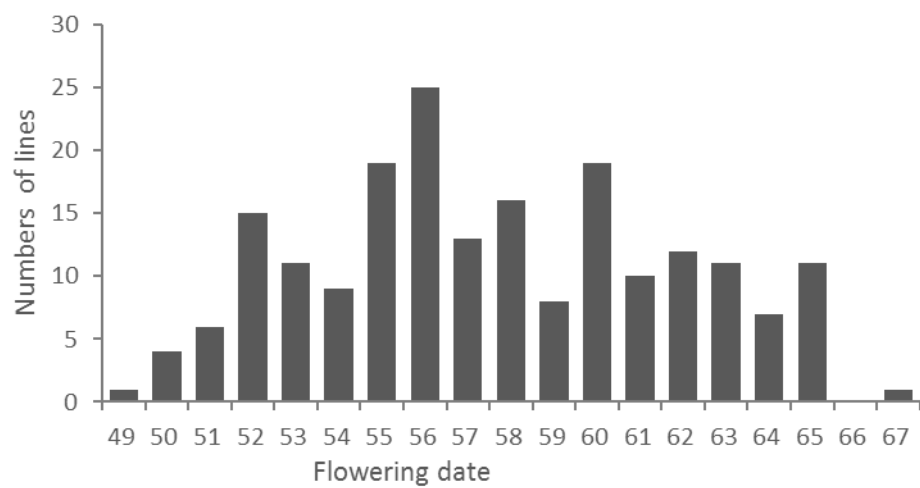

B 2013

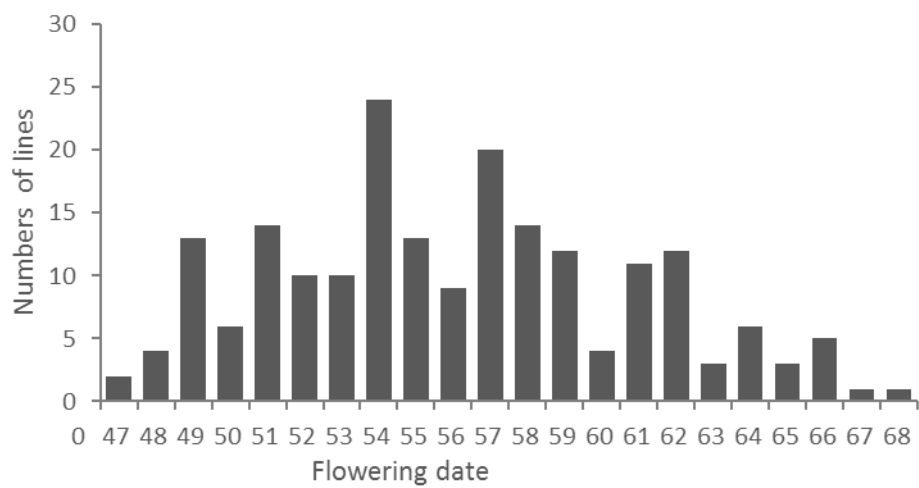

Supplement: S2 Fig — Frequency distributions of days to flowering for the recombinant-inbred lines derived from a cross between Glycine soja and ‘Fukuyutaka’ in 2012 (A) and 2013 (B). Days to flowering for ‘Fukuyutaka’ were 47 and 46 in 2012 and 2013, respectively. Days to flowering for G. soja were 67 and 69 in 2012 and 2013, respectively. (PDF) [file pone.0189440.s002.pdf]
